# Distinct cognitive effects and underlying transcriptome changes upon inhibition of individual miRNAs in hippocampal neurons

**DOI:** 10.1038/srep19879

**Published:** 2016-01-27

**Authors:** Josephine Malmevik, Rebecca Petri, Pina Knauff, Per Ludvik Brattås, Malin Åkerblom, Johan Jakobsson

**Affiliations:** 1 Department of Experimental Medical Science, Wallenberg Neuroscience Center and Lund Stem Cell Center, BMC A11, Lund University, Sölvegatan 17, 221 84 Lund, Sweden

## Abstract

MicroRNAs (miRNA) are small, non-coding RNAs mediating post-transcriptional regulation of gene expression. miRNAs have recently been implicated in hippocampus-dependent functions such as learning and memory, although the roles of individual miRNAs in these processes remain largely unknown. Here, we achieved stable inhibition using AAV-delivered miRNA sponges of individual, highly expressed and brain-enriched miRNAs; miR-124, miR-9 and miR-34, in hippocampal neurons. Molecular and cognitive studies revealed a role for miR-124 in learning and memory. Inhibition of miR-124 resulted in an enhanced spatial learning and working memory capacity, potentially through altered levels of genes linked to synaptic plasticity and neuronal transmission. In contrast, inhibition of miR-9 or miR-34 led to a decreased capacity of spatial learning and of reference memory, respectively. On a molecular level, miR-9 inhibition resulted in altered expression of genes related to cell adhesion, endocytosis and cell death, while miR-34 inhibition caused transcriptome changes linked to neuroactive ligand-receptor transduction and cell communication. In summary, this study establishes distinct roles for individual miRNAs in hippocampal function.

MicroRNAs (miRNAs) are small non-coding RNAs with distinct expression patterns throughout the body, including parts of the brain^1^. miRNAs cause the degradation and/or inhibited translation of specific target messenger RNAs (mRNAs) via complementary base pairing in the RNA-induced silencing complex; RISC^2^,^3^. Each miRNA contains seed sequences necessary for the recognition and the binding of target mRNAs. Seed sequences are generally situated at positions 2–7 from the 5′ end of the miRNA and are conserved within a miRNA family.

In recent years, miRNAs have been implicated in the generation, survival, branching, excitation and plasticity of neuronal cells^1^,^4^,^5^. Interestingly, inhibition of the miRNA biogenesis pathway through conditional deletion of the Dicer enzyme in the adult rodent forebrain, results in an increased cognitive capacity, suggesting a role for miRNA regulation in learning and memory^6^. However, both Dicer and Argonaute 2 (AGO2) have functions unrelated to the miRNA biogenesis pathway^9^, and thus, it has not been fully asserted that miRNAs are accountable for the observed cognitive and degenerative phenotypes. Prolonged deletion of Dicer in specific neuronal populations, including cells of the hippocampus, leads to neuronal cell death, thus implicating miRNA involvement in neuronal survival^6^,^7^. In a recent study, we analysed the mRNAs that are targeted by miRNAs in hippocampal neurons of the adult mouse hippocampus, using AGO2-RNA-interacting protein immunoprecipitation (AGO2-RIP) followed by next generation sequencing. AGO2 is a part of the RISC, with crucial roles in the miRNA-mediated degradation of targeted mRNAs. Using AGO2-RIP, we identified 2177 potential target mRNAs in the RISC, regulating key neuronal functions such as cell signalling, transcription and axon guidance^8^.

More than 2500 miRNAs in human and 1900 miRNAs in mouse have been identified (*hsa* and *mmu* mature names in miRbase, accessed 24/4/2015; Kozomara and Griffiths-Jones 2014). Since their discovery, individual miRNAs have been linked to specific functions in different tissues and developmental stages. This data has been collected through techniques including, but not limited to, fluorescent *in situ* hybridization, green fluorescent protein (GFP) sensor vectors, miRNA arrays and RNA sequencing^10^,^11^,^12^. Still, the function of most individual miRNAs in distinct regions of the central nervous system (CNS) remains poorly characterised. In this study, we analyse the functional role of three miRNAs that are known to be expressed at high levels in the CNS, and have been implicated in hippocampal function; miR-124, miR-9 and miR-34^13^,^14^.

miR-124 is a highly conserved miRNA^15^, and is represented by three genomic loci; mir-124-1, mir-124-2 and mir-124-3^16^. Being one of the most highly expressed miRNAs in the hippocampus^13^, miR-124 has a prominent activity in neurons, starting in early neuronal differentiation^15^,^17^. With respect to disease and degeneration, miR-124 is reduced in ischemia and is indirectly linked to the progression of glioma^18^,^19^. Furthermore, decreased levels of miR-124 have been shown to reduce maturation and survival of neurons in the dentate gyrus, whereas increased levels of miR-124 has rescued behavioural deficits in frontotemporal dementia^20^,^21^. Altogether, miR-124 has been linked to various neuroprotective and degenerating roles^18^,^19^,^20^,^21^.

miR-9 is known to be involved in neuronal differentiation^22^,^23^, axonal extension and branching^24^. There are three homologous members of the miR-9 family (mir-9-1, mir-9-2, mir-9-3), originating from three different genomic loci in mouse^16^, with high conservation among species^25^. The miR-9 family is enriched in the brain, with high abundance in neurogenic regions of the adult brain such as the hippocampus^13^,^25^. It is highly expressed in neural precursor cells and also at lower levels in mature neurons^26^. Using miR-9 sensor mice, we have previously demonstrated miR-9 activity in all CNS cell types throughout the brain except microglia and a few, smaller neuronal populations in the septum, habenula and hypothalamus^27^.

Another highly expressed miRNA family in the brain with enrichment in the hippocampus is the miR-34 family^13^,^14^. This family is highly conserved^15^ and expressed as three members in mouse; miR-34a, miR-34b and miR-34c, originating from two precursor transcripts^16^. miR-34 has been linked to neurogenesis, spine morphology, neurite outgrowth, neurodegeneration as well as hippocampal memory formation^14^,^28^,^29^.

In the present study, we analysed changes in the transcriptome after individual inhibition of miR-9, miR-34 and miR-124 using AAV-delivered miRNA sponges^30^,^31^,^32^. The inhibition of each miRNA was investigated with respect to transcriptional changes and behavioural phenotypes. Our data demonstrate distinct changes in the hippocampal transcriptome after delivery of miRNA sponges, accompanied by specific effects on cognition by different miRNAs. This study underlines the importance of individual miRNAs in the function of mouse hippocampal neurons.

## Results

### miR-124, miR-9 and miR-34 are active in adult mouse hippocampal neurons

**Figure 1:**
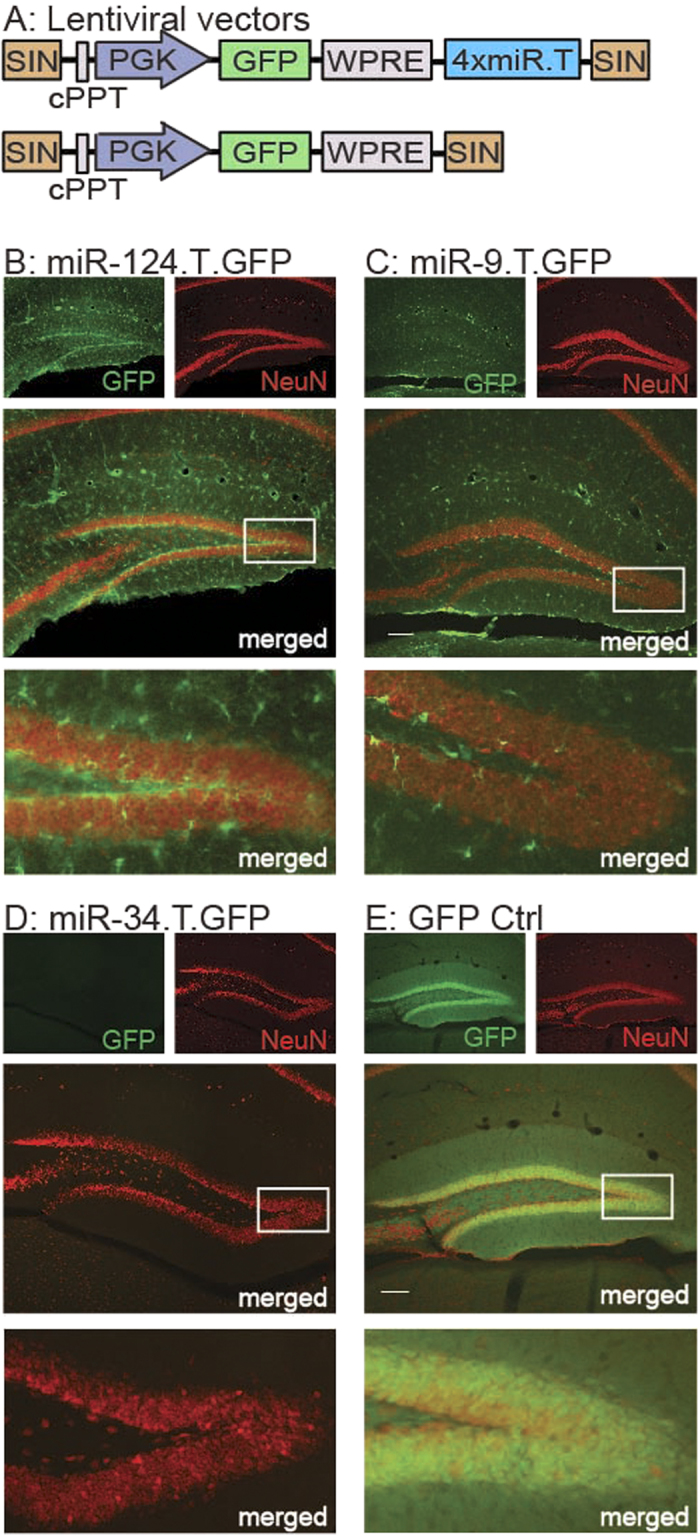
Activity of three miRNAs in hippocampal neurons. miRNA activity was visualised in transgenic mice using a negative GFP reporter system incorporating perfectly complementary binding sites for each respective miRNA. (**A**) Expression of the GFP reporter was achieved under the regulation of a PGK promoter with (upper panel) or without (lower panel; GFP Ctrl) the inclusion of four perfectly complementary target sites (miR.T) for a specific miRNA, separated by a WPRE element in a self-integrating (SIN) lentiviral vector. Lentiviral transgenesis resulted in integration of these vectors in all cell types. B-D) The presence of four perfectly complementary target sites for either miR-124 (**B**) miR-9 (**C**) or miR-34 (**D**), limited GFP expression to a subpopulation of cells or in no cells at all, indicating activity of each respective miRNA in GFP-negative cells. Hippocampal neurons were GFP-negative in miR-124.T.GFP, miR-9.T.GFP and miR-34.T.GFP mice, indicating the activity of each respective miRNA in these cells. miR-124.T.GFP mice displayed GFP expression, i.e. lack of miR-124 activity, in astrocytes and microglia (see Akerblom *et al*. 2012a), whereas only microglia had this characteristic in miR-9.T.GFP mice (see Akerblom *et al.* 2013). E) In GFP Ctrl transgenic mice, any cell type, including hippocampal neurons of the dentate gyrus, can express GFP. Scale bars in merged middle panels are 100 μm.

miR-124, miR-9 and miR-34 are known to be highly expressed in the mouse hippocampus^13^. We have also demonstrated the activity of miR-124 and miR-9 in the adult mouse brain using transgenic miR.T.GFP sensor mice^17^,^27^. These transgenic miR.T.GFP mice are designed to express a GFP transcript that also contains four identical and perfectly complementary target sequences (miR.T) for a specific miRNA family (Fig. 1A, upper panel). The transgenic design of the miR.T.GFP mice provides a negative, indirect GFP reporter sensor system linked to the miRNA target sequence. Thus, the cellular presence of a miRNA with binding capacity to the target sequences will lead to the inhibition of GFP transgene expression. On the contrary, cells lacking the miRNA will express GFP. We and others have previously demonstrated this approach as an efficient method to study miRNA activity pattern *in vivo* at a cellular resolution^12^,^17^.

In the current study, we took advantage of previously published miR-124.T.GFP and miR-9.T.GFP mice^17^,^27^, and moreover generated miR-34.T.GFP mice, to analyse the activity of these individual miRNAs in the mouse hippocampus in more detail. In line with our previous reports on miR-124.T.GFP mice, all hippocampal neurons were GFP negative due to the endogenous activity of miR-124, while glial cell types expressed GFP (Fig. 1B). Thus, miR-124 is a neuron-specific miRNA expressed in all hippocampal neurons. Likewise, hippocampal neurons do not express GFP in miR-9.T.GFP mice, demonstrating the activity of miR-9 in these cells (Fig. 1C). We have previously demonstrated activity of miR-9 in all CNS cell types except microglia and a few, smaller neuronal populations outside the hippocampus^27^. With regards to miR-34.T.GFP (Fig. 1D), no cells of the hippocampus express GFP, indicating that this miRNA family is active in all cell types of this brain region. We have also generated GFP Ctrl mice, which lack miRNA target sequences and ubiquitously express GFP in all cells (Fig. 1A, lower panel; Fig. 1E). Taken together, these data show that miR-124, miR-9 and miR-34 are active in hippocampal neurons. Furthermore, while miR-124 is neuron-specific, miR-9 and miR-34 are also active in certain glial populations.

### Injection of AAV sponge vectors into the mouse hippocampus

**Figure 2:**
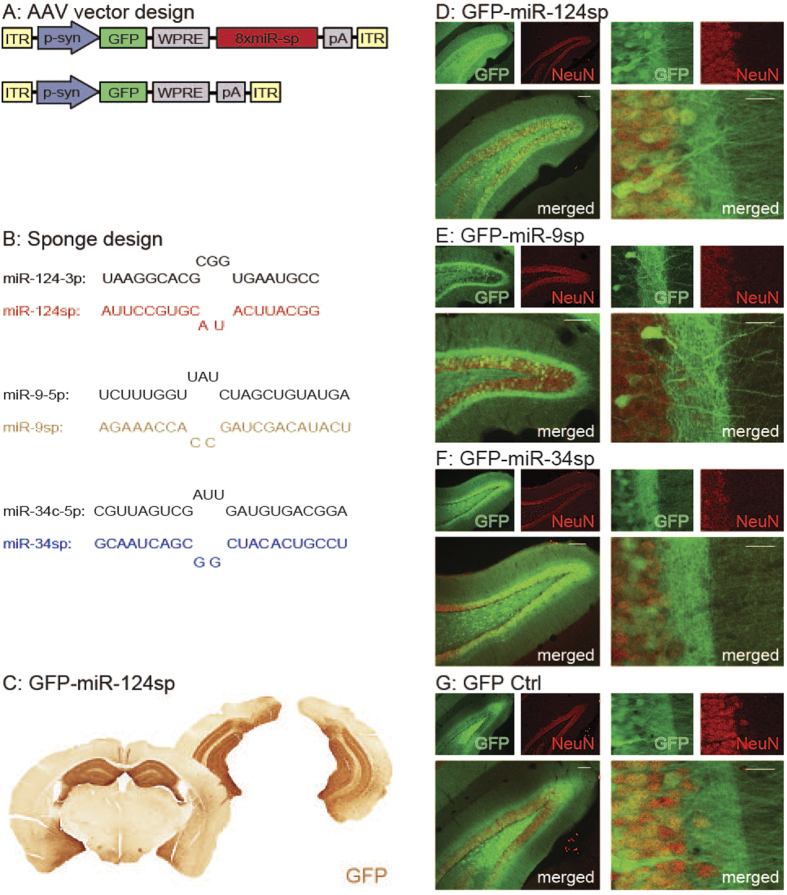
miRNA inhibition using AAV-miRNA sponges. (**A**) AAV-miRNA sponges were expressed in order to inhibit individual miRNA families. These vectors express a GFP reporter under the influence of a synapsin promoter (p-syn) with (upper panel) or without (lower panel; GFP Ctrl) the presence of eight imperfectly complementary target sites for a specific miRNA, all within inverted terminal repeats (ITRs) of an AAV2/5 vector. (**B**) The design of the miR-124sp, miR-9sp and miR-34sp sequences in comparison to the endogenous miRNA. (**C–G**). (**C**) DAB immunohistochemistry, (**D–G**) fluorescence immunohistochemistry. Injection of AAV2/5 pseudotyped vectors into the hippocampus (three locations per hemisphere), resulted in the expression of the GFP reporter in a majority of hippocampal neurons of the dentate gyrus in the presence of a miR-124sp sequence (**C**–**D**), a miR-9sp sequence (**E**), a miR-34sp sequence (**F**), or without such a sequence (**G**). Scale bars 100 μm (**D**–**G**); (left merged panels) and 20 μm (**D**–**G**); (right merged panels).

Following the determination of miRNA activity in hippocampal neurons of transgenic GFP sensor mice, we further analysed the inhibition of each respective miRNA in wild-type mice. In order to inhibit individual miRNA families in the neurons of the adult mouse hippocampus, we used AAV vectors expressing miRNA sponges (Fig. 2A,B). These sponges consist of eight imperfectly complementary sequences (miR-sp) to the seed sequence of a specific miRNA family. Through this imperfect complementary binding, the resulting bulge of the sponge inhibits its own degradation by the miRNA. Thus, this sequestration prevents the miRNAs from binding their endogenous target mRNAs, while keeping the sponge intact and only causing a marginal decrease in miRNA levels^8^,^17^,^32^.

Bilateral injections of synapsin-driven AAV sponge vectors into three locations of the hippocampus of wild-type adult male C57BL/6 mice (see coordinates in Methods), achieved neuron-specific expression in a majority of hippocampal neurons. For visualisation purposes, the AAV vectors also contained a GFP reporter upstream of the sponge sequences. Eight weeks post injection, we detected GFP expression in neurons throughout the hippocampus with an emphasis on the dentate gyrus. Injections of GFP-miR-124sp (n = 18; Fig. 2B, upper panel; Fig. 2C,D), GFP-miR-9sp (n = 20; Fig. 2B, middle panel; Fig. 2E) and GFP-miR-34sp (n = 12; Fig. 2B, lower panel; Fig. 2F) all resulted in widespread high-level GFP expression in neuronal cells throughout the hippocampus. An AAV-GFP vector without sponge sequence was used as a control in each experiment (Fig. 2A, lower panel; Fig. 2G)

### Sponges mediate increased expression of target genes

**Figure 3:**
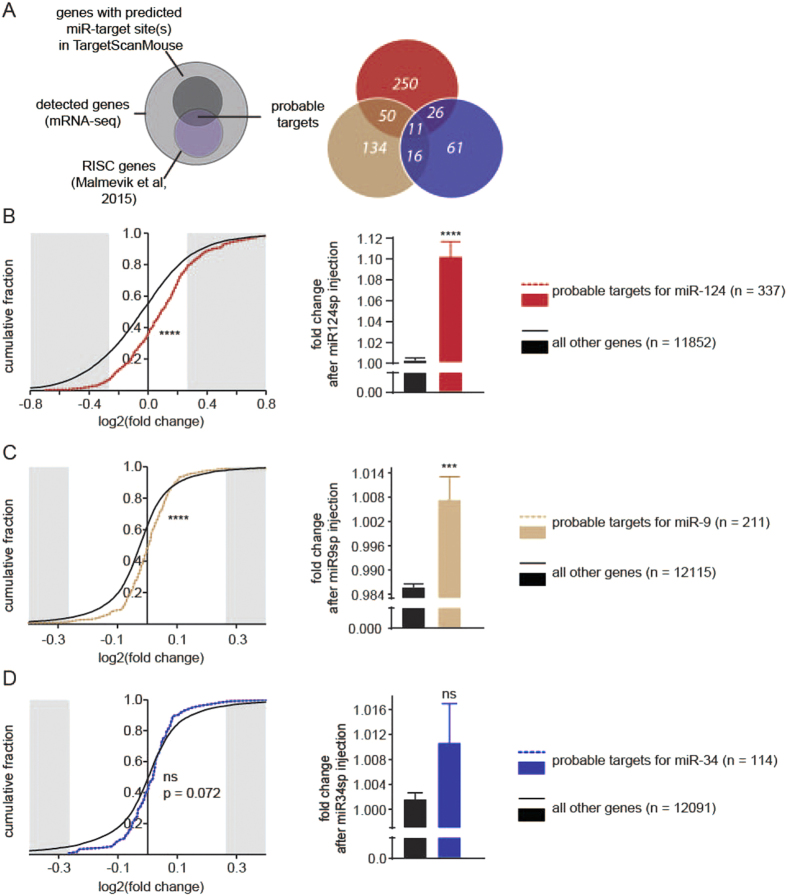
Transcriptome changes of probable targets after miRNA inhibition. (**A**) In the list of all detected genes using mRNA-seq after each respective miRNA inhibition (light grey), RISC genes (light purple; Malmevik *et al.* 2015) with at least one computationally predicted and evolutionary conserved target site for this miRNA (miR-target site(s); dark grey; TargetScanMouse 6.2) were identified as probable targets (overlapping area, left panel). Some probable targets were shared between the three analysed miRNAs (right panel). Each subset of probable targets was compared to all other genes in each respective miRNA sponge (miR-sp) experiment. (**B**) After miR-124 inhibition, this subset (n = 337, red/dotted, left panel) had a significantly different cumulative fraction distribution of its log2(fold change in miR-124sp/GFP Ctrl) in comparison to that of all other genes (n = 11852, black; ****p < 0.0001, Kolmogorov-Smirnov Z test), demonstrating up-regulated expression of miR-124 target genes. The genes of this subset (red bar, right panel) also had an overall higher average fold change after miR-124sp injection in comparison to all other genes (black bar; ****p < 0.0001, unpaired parametric two-tailed t-test). (**C**) Similarly, the equivalent subset of probable targets of miR-9 (n = 211; brown/dotted) had a significantly different fold change after miR-9sp inhibition in comparison to all other genes (n = 12115, black), as shown in a log2(fold change) cumulative fraction graph (**C**, left panel; ****p < 0.0001, Kolmogorov-Smirnov Z test) and in an overall fold change bar chart (**C**) right panel; ***p < 0.001, unpaired parametric two-tailed t-test). (**D**) The equivalent subset of probable targets of miR-34 (n = 114; blue/dotted) after miR-34sp inhibition exhibited the same trend as miR-124 and miR-9 in comparison to all other genes (n = 12091, black), however this did not reach significance. FC > 1.2 and FC < 1/1.2 are presented as shaded grey areas of each respective graph.

The effects of AAV sponge expression were investigated on a molecular level by conducting mRNA-sequencing (mRNA-seq) on RNA from entire hippocampi. In order to confirm that the sponges act on the targeted miRNAs, we defined a subset of genes that were highly probable targets for each miRNA family and investigated transcriptional changes in these genes. These subsets were based on our recently published data of 2177 genes that are present in the RISC of adult mouse hippocampal neurons^8^. This miRNA targetome of 2177 genes has been identified through AAV-mediated expression of a known component of the RISC; AGO2, in a GFP-AGO2 fusion protein. The synapsin-driven expression of this GFP-AGO2 fusion protein in the mouse hippocampus allowed for the analysis of RISC-associated RNAs specifically from hippocampal neurons through GFP-targeted RIP and subsequent RNA sequencing^8^. Out of these 2177 RISC genes in hippocampal neurons, we selected probable targets of each miRNA based on computationally predicted and evolutionary conserved miR-target sites (TargetScanMouse 6.2; Fig. 3A, left panel). These probable targets accounted for 18.4% (miR-124; 337 genes with predicted miR-124 target sites out of 1830 detected RISC genes), 10.6% (miR-9; 211 genes out of 1991 detected RISC genes) and 6.2% (miR-34; 114 out of 1837 detected RISC genes) of all detected AGO2-bound genes in each experiment^8^. Furthermore, a proportion of probable targets was shared between the three miRNAs (Fig. 3A, right panel).

In cumulative fraction graphs (y-axis), we plotted the fold change of each gene after the injection of sponge vectors (x-axis). After miR-124 inhibition, the cumulative distribution of the fold change values for probable miR-124-targets was significantly different in comparison to all other genes indicating an overall higher expression level (Fig. 3B, left panel). In addition, this subset also had an overall higher average fold change after miR-124sp injection in comparison to all other genes (Fig. 3B, right panel). Similarly, the probable miR-9-targets had a significantly different cumulative distribution after miR-9sp injection, indicating increased expression and an overall higher average fold change in comparison to all other genes (Fig. 3C). A similar trend was also observed for the probable miR-34 targets after miR-34 inhibition, although this difference did not reach significance (Fig. 3D). Taken together, these data demonstrate a global and specific up-regulation of miRNA targets depending on the miRNA being inhibited, thereby indicating sponge vector functionality. Thus, AAV-miRNA sponges are efficient and versatile tools for loss-of-function studies of individual miRNA families in the brain.

### Distinct transcriptome changes after inhibiting individual miRNAs

**Figure 4:**
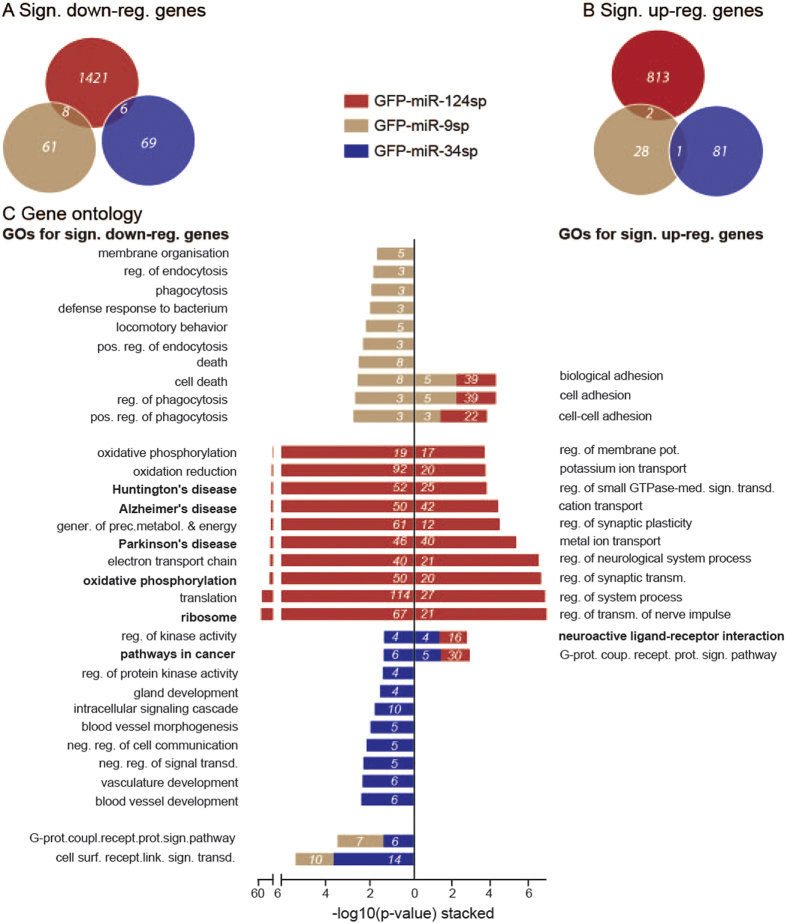
Specific transcriptome changes after inhibition of each miRNA. (**A**) Out of all detected genes in the total mRNA-seq, 1435, 69 and 75 genes were significantly down-regulated after miR-124, miR-9 and miR-34 inhibition, respectively. (**B**) For each of these three miRNA families, 815, 31 and 82 genes were significantly up-regulated. Very little overlap existed between the different miRNA inhibitions. (**C**) DAVID gene ontology analysis was performed on genes that were significantly down-regulated (left side) and up-regulated (right side) in each miRNA sponge experiment, showing the -log10(p-value) significance of each GO term (GO_BP, or KEGG pathway in bold) together with the total number of members enriched in this GO term (white text on each bar). All, or the top 10, GO terms are included and also any shared significant GO terms between the experiments (stacked bars).

In order to further analyse transcriptome changes after miRNA sponge delivery, we analysed all significantly altered genes in each miRNA inhibition experiment, irrespective of being probable targets (Fig. 4). As an example, miR-124 inhibition (red) resulted in significant (p < 0.05) down-regulation of 1435 genes (Fig. 4A; 1420 + 8 + 6 = 1435; fold change: FC < 0.833) and up-regulation of 815 genes (Fig. 4B; 813 + 2 = 815; FC > 1.2). The up-regulated gene list consisted of 20% (161 genes) previously validated miR-124 target genes (miRwalk and tarbase, see Methods; see Supplementary Table S1).

Out of the approximately twelve thousand detected genes in mRNA-seq of each miRNA inhibition experiment, 18.5% (miR-124; 2249 significantly altered genes out of 12189 detected genes in mRNA-seq), 0.81% (miR-9; 100 out of 12326 detected genes) and 1.29% (miR-34; 157 out of 12205 detected genes) were significantly altered after inhibition of each miRNA. Separate comparisons of down-regulated and up-regulated genes between each sponge experiment demonstrated distinct transcriptome changes with minimal overlap between each miRNA (Fig. 4A,B). This underlines individual effects of each miRNA in adult mouse hippocampal neurons.

The function of the significantly altered genes in each miRNA sponge experiment were further analysed using DAVID gene ontology analysis, with respect to GO_BP and KEGG pathways (Fig. 4C). miR-124 inhibition (red bars) resulted in significant up-regulation of genes (n = 815; Fig 4C, right) involved in 114 GO_BP and KEGG pathways, including synaptic plasticity and transmission of nerve impulse in the top 10 most significant terms. Significantly down-regulated genes (n = 1435; Fig. 4C, left) were implicated in 68 GO_BPs and KEGG pathways, including the ribosome and in neurodegenerative diseases such as Huntington’s, Alzheimer’s, and Parkinson’s disease in the top 10 most significant terms. miR-9 inhibition (brown bars) resulted in an up-regulation of 31 genes involved in 3 GO_BPs and KEGG pathways related to cell adhesion. Conversely, significantly down-regulated genes (n = 69) after miR-9 inhibition were enriched for 20 GO_BPs and KEGG pathways, including endocytosis, phagocytosis and cell death in the top 10 most significant terms. The inhibition of miR-34 led to significant up-regulation of 82 genes enriched in 2 GO_BPs; neuroactive ligand-receptor interaction and the G-protein coupled receptor protein signalling pathway. Conversely, miR-34 inhibition resulted in significant down-regulation of 75 genes involved 15 GO BPs and KEGG pathways, including regulation of signal transduction, cell communication and pathways in cancer in the top 10 most significant terms. Comparison of any pair of miRNA sponge experiments showed only shared enrichment of a few GO_BPs and KEGG pathways (Fig. 4C, stacked bars). For example, significantly up-regulated genes after inhibition of miR-124 and miR-9 were both enriched for the GO terms biological adhesion, cell adhesion and cell-cell adhesion (Fig. 4C, brown and red stacked bars on right). However, different transcripts were responsible for this enrichment, showing the distinct transcriptome changes caused by inhibition of each miRNA.

### Pathway analysis underlines distinct transcriptome changes after inhibition of individual miRNAs

**Figure 5:**
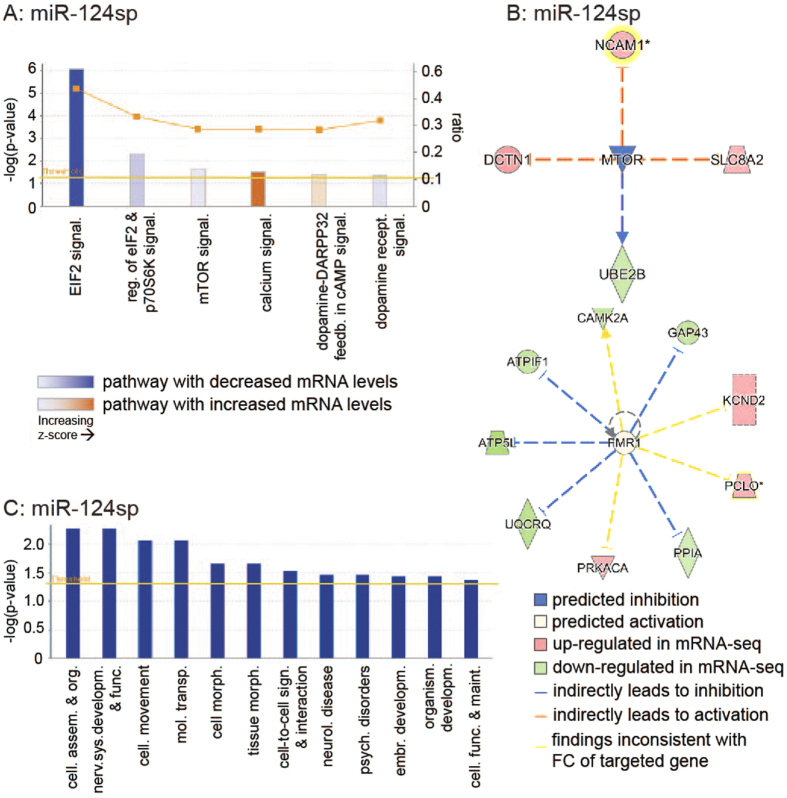
Ingenuity Pathway Analysis of transcriptome changes after miR-124 inhibition. miR-124 inhibition resulted in significantly altered levels of mRNAs enriched in (**A**) canonical pathways, (**B**) upstream regulators (**C**) and disease & biological functions. (**A**) miR-124sp injection led to differential expression of mRNAs involved in EIF2-, calcium- and mammalian target of Rapamycin (mTOR) signalling with each respective z-score, ratio and –log(p-value). The calculated z-score indicates a pathway with genes exhibiting overall increased mRNA levels (orange bars) or decreased mRNA levels (blue bars). The ratio (orange dots connected by a line) indicates the ratio of genes from the dataset that map to the pathway divided by the total number of genes that map to the same pathway, e.g. >40% for EIF2 signalling. (**B**) Potential upstream regulators identified in the miR-124sp dataset included mTOR (upper panel) and Fragile X mental retardation 1 (FMR1; lower panel). mTOR was predicted as an inhibited upstream regulator (blue; Fischer’s Exact test, *p = 0.0389), potentially causing indirect (dashed lines) activation of three mRNAs. The inhibition of mTOR was also predicted to have caused lowered activity of one mRNA. Genes were highlighted (yellow aura) when multiple transcripts were present in the dataset, upon which the maximum absolute fold change (FC) value of any one transcript was visualised. FMR1 was predicted to be an enhanced upstream regulator (lower panel; Fischer’s Exact test, *p = 0.047). The predicted activation of FMR1 was thought to result in indirect inhibition of five mRNAs. Four mRNAs were believed to be either inhibited or enhanced by FMR1 but displayed inconsistent findings in fold change after miR-124 inhibition (yellow dashed lines). (**C**) The IPA disease/function analysis confirmed that gene level alterations in miR-124sp-injected mice corresponded to neurological disease, nervous system development and function, and cell-to-cell signalling and interaction, among others. Threshold levels in (**A,C**) are based on significance (p = 0.05).

Analysis of up- and down-regulated genes after miRNA inhibition was also performed with respect to canonical pathways, upstream analysis and disease/function analysis using Ingenuity Pathway Analysis (IPA; see more details in Methods; see Supplementary Table S2A-C). In particular, we focused our additional investigation on the transcriptome changes observed after miR-124 inhibition. miR-124sp expression resulted in significantly altered levels of mRNAs that were enriched in canonical pathways such as EIF2-, calcium- and mammalian target of Rapamycin (mTOR) signalling (Fig. 5A). Upstream analysis of observed transcriptome changes further identified effects on two specific genes; inhibited function of mTOR (Fig. 5B, upper panel) and activated function of Fragile X mental retardation 1 (FMR1; Fig. 5B, lower panel). mTOR has been strongly linked to cellular growth and synaptic plasticity^33^, and FMR1 is essential for cognitive brain development^34^. FMR1 contains a partial predicted target site for miR-124, which may contribute to the activation of FMR1 function upon miR-124 inhibition (TargetScan Mouse 6.2). mTOR does not contain any target sites for miR-124, and is therefore most likely inhibited as a secondary effect. With regards to disease/functional analysis, miR-124sp-injected mice exhibited transcriptional changes in mRNAs involved in nervous system development and function, neurological disease, cell-to-cell signalling and interaction, and psychological disorders (Fig. 5C). Comparison of the three miRNA sponge experiments demonstrated distinct patterns of altered canonical pathways (see Supplementary Table S2A), upstream regulators (see Supplementary Table S2B) and diseases & biological functions (see Supplementary Table S2C). Thus, this pathway analysis further underlines the distinct effect of three individual miRNAs on the function of hippocampal neurons.

To validate the accuracy and reproducibility of the mRNA-seq data set, we performed a microarray analysis on miR-124sp RNA samples. Analysis of the microarray data showed a good correlation between up- and down-regulated genes using the two techniques (see Supplementary Fig. S1A, compare top and bottom panels). Furthermore, comparison of gene ontology analysis in the significantly altered genes of each respective analysis showed 8 of 10 exact matches of GO_BPs and KEGG pathways (see Supplementary Fig. S1B, compare top and bottom panels). These data confirm that mRNA-seq is a robust technique to study transcriptome changes following miRNA inhibition in mouse hippocampus.

### Inhibition of individual miRNAs in the hippocampus affects cognition

**Figure 6:**
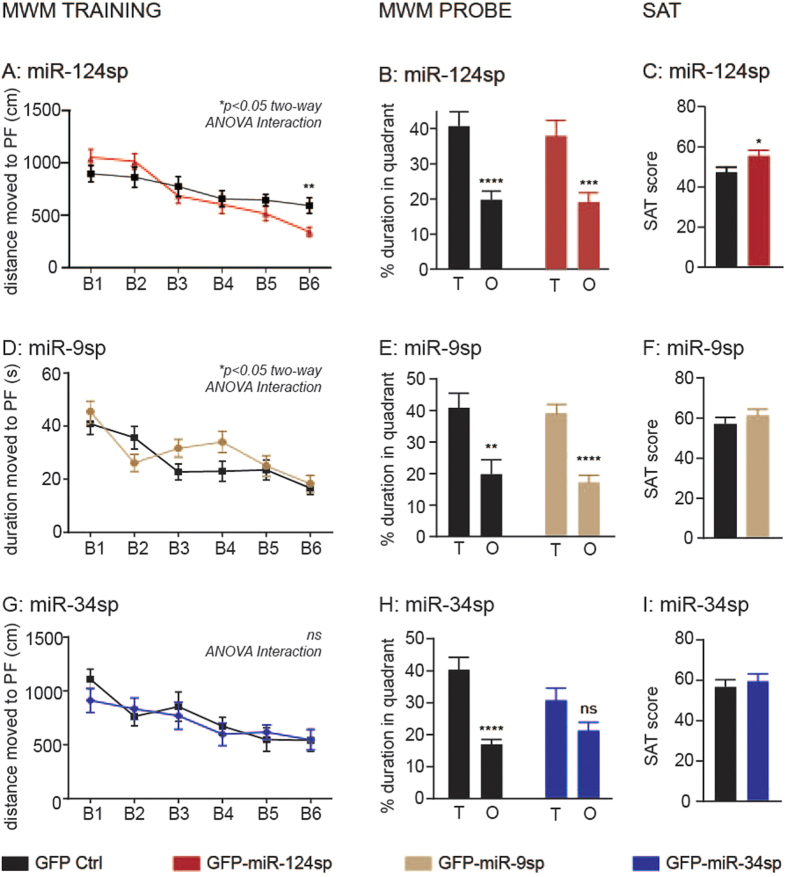
Specific cognitive effects after inhibition of each miRNA. (**A**) In a Morris Water Maze (MWM) test, miR-124sp mice swam significantly shorter distances over the paired trials (block 1 through 6; B1–B6) in order to find the hidden platform (PF; *p < 0.05 repeated measures two-way ANOVA with Sidak’s multiple comparisons test; Interaction between vector type and block). This was particularly evident in B6 (**p < 0.01; unpaired parametric two-tailed t-test). (**B**) The two experimental groups performed equally well in a MWM probe test (GFP Ctrl ****p < 0.0001; miR-124sp ***p < 0.001; unpaired parametric two-tailed t-tests). (**C**) In a spontaneous alternation task (SAT) test, miR-124sp-injected mice alternated between the arms of a symmetric plus maze to a larger extent than GFP Ctrls (*p < 0.05, unpaired parametric two-tailed t-test). (**D**) miR-9 inhibition resulted in impaired learning in the MWM training phase, where miR-9sp-injected mice learnt the task of finding the hidden platform differently from GFP Ctrl (*p < 0.05, repeated-measures two-way ANOVA with Sidak’s multiple comparisons test; Interaction between vector type and block). (**E**) There was no difference between the experimental groups in the MWM probe test, where both groups learnt the task (GFP Ctrl **p < 0.01; miR-9sp ****p < 0.0001; unpaired parametric two-tailed t-tests). (**F**) No significant difference was found in the SAT test between the groups. (**G**) miR-34 inhibition had no effect upon the training phase of the MWM. (**H**) However, while the GFP Ctrl mice learnt the MWM probe test (****p < 0.0001, unpaired parametric two-tailed t-test), the miR-34sp-injected group did not (ns p = 0.0522, unpaired parametric two-tailed t-test), indicating an impaired reference memory. (**I**) No significant difference was detected between the two groups in the SAT test.

To study the role of the three miRNAs in hippocampus-dependent behaviour, we conducted a battery of behavioural tests seven to eight weeks after AAV-sponge injection. Interestingly, all groups injected with miRNA sponges exhibited an altered cognitive phenotype in the Morris Water Maze (MWM) task of spatial learning and memory in comparison to each corresponding GFP Ctrl group (Fig. 6). Over three consecutive training days with two blocks (B = paired trial averages) each, miR-124sp-injected mice displayed a different learning pattern in comparison to GFP Ctrls (Fig. 6A). miR-124sp injected mice learned to swim significantly shorter distances than GFP Ctrls in order to find the hidden platform (PF) in opaque water using specific visual cues for orientation. In B1, miR-124sp-injected mice and GFP Ctrls swam 1051+/− 331 cm and 895+/− 334 cm, respectively (mean+/− standard deviation). At B6, the equivalent values were 340.6+/− 196cm and 592+/− 313 cm for the two groups, respectively. No difference was observed between the two groups in a probe trial (Fig. 6B) or in a visible platform test (see Supplementary Fig. S2A). To verify the improved learning of the miR-124sp mice, we adapted a spontaneous alternation task (SAT) test^35^,^36^. In this symmetrical walled plus-shaped maze, we analysed the spontaneous alternation of the mice between the four arms during 10 min of unimpeded exploration. A SAT score was calculated, taking into account the arm choices in succession together with the total number of entries. This SAT score serves as a measure of working memory capacity. In line with our previous findings in the MWM, miR-124sp-injected mice received significantly higher SAT scores than GFP Ctrls, indicating an enhanced working memory (Fig. 6C). The total number of arm entries was not significantly different when comparing the two groups (see Supplementary Fig. S2BM). In the Open Field test of general activity and in the Elevated plus maze test of anxiety-like behaviours, no difference was detected between miR-124sp-injected mice and GFP Ctrls (see Supplementary Fig. S2C–D). In summary, miR-124sp-injected mice exhibited improved learning and working memory in comparison to GFP Ctrls, without any effect on general activity or anxiety-related behaviours.

Behaviour was also analysed after injection of the miR-9 sponge. In contrast to miR-124sp-injected mice, miR-9sp-injected mice did not learn to find the location of the MWM PF as quickly as GFP Ctrls, thus implicating impaired learning in the MWM training phase (Fig. 6D; n = 20). The experimental groups performed equally well in the subsequent MWM probe test (Fig. 6E), the MWM visible platform test (see Supplementary Fig. S2E), the SAT task (Fig. 6F; see Supplementary Fig. S2F), and in tests of general activity (Open Field; n = 8; see Supplementary Fig. S2G) and anxiety-like behaviour (Elevated plus maze; n = 20, see Supplementary Fig. S2H).

In contrast to miR-124 and miR-9 inhibition, miR-34sp injection did not affect performance during the MWM training phase (Fig. 6G). However, in the subsequent MWM probe test, where the platform had been removed from the arena, mice with inhibited miR-34 function did not spend a significant proportion of time in the target (T) quadrant in comparison to the opposite (O) quadrant (Fig. 6H). Thus, these mice displayed an impaired capacity to retain reference memory. No difference was detected between the two experimental groups with regards to the MWM visible platform test (see Supplementary Fig. S2I) or short-term working memory in the SAT task (Fig. 6I; see Supplementary Fig. S2J). Behavioural testing also showed equal levels of general activity and anxiety-like behaviour (see Supplementary Fig. S2K-L).

## Discussion

In this study, we demonstrate the use of AAV-delivered miRNA sponges to inhibit the activity of specific miRNAs in neurons of the adult mouse hippocampus. We and others have previously demonstrated that miRNA sponges can inhibit miRNA function *in vivo*, including studies in the brain^12^,^17^,^30^,^31^,^32^,^37^. In this study, we demonstrate global transcriptome changes caused by miRNA sponges, including a general up-regulation of predicted probable targets for the inhibited miRNAs. Furthermore, the inhibition of the three individual miRNAs; miR-124, miR-9 and miR-34, led to distinct transcriptome changes with minimal overlap between the different miRNAs. Thus, AAV-delivered sponges are a versatile tool to study miRNA function in the adult brain.

A major hurdle in the study of miRNAs is the identification of direct target genes in different tissues. In this study, we took advantage of a previous data set^8^ identifying transcripts bound to AGO2 in hippocampal neurons and thus likely to be direct targets of miRNAs. By combining this gene list with predicted, conserved target sites (TargetScan) we generated separate lists with high-confidence probable targets for miR-124, miR-9 and miR-34, respectively. We found that high-confidence probable targets were up-regulated in cells of the hippocampus upon AAV-sponge injection. This up-regulation was modest and on average represented a 10% increase in mRNA levels. Although this small increase may be partially explained by technical issues, such as the transcriptome analysis of bulk tissue rather than only analysing mRNA in neurons, and the in-complete neuronal targeting of the AAV vector, the modest up-regulation is line with what has been previously reported^2^,^5^. Similarly to the modest transcriptional regulation, we also detected mild but significant phenotypes in behavioural tests, indicating that each miRNA modulates behaviour in a distinct manner. Taken together, these observations strengthen the idea that each miRNA form parts of large gene networks, in which it impacts on a low scale per gene, and with a greater impact on a genome-wide level.

The inhibition of miR-124 led to vast transcriptome changes and improved performance in the MWM task and the SAT test. These findings suggest that inhibition of miR-124 in hippocampal neurons results in a similar cognitive enhancement as obtained after Dicer deletion in the adult forebrain^6^. Thus, miR-124 play an important role in regulating hippocampal spatial learning and memory function. miR-124 is highly expressed in mouse hippocampus, accounting for 15% of all miRNAs in this brain region, as determined using RNA-seq^13^. In addition, we have previously shown that miR-124 targets hundreds of genes in hippocampal neurons^8^. These miR-124 targets were highly enriched for genes involved in transcription, which is likely to cause large-scale downstream transcriptional changes.

In the present study, we performed a detailed transcriptome analysis in hippocampal neurons after miR-124 inhibition. We found up-regulation of genes involved in synaptic plasticity and in the regulation of transmission of nerve impulse. These transcriptional changes fit well with the observed behavioural phenotype of miR-124sp-injected mice and implicate dysregulation of miR-124 in both psychiatric and neurodegenerative disorders. Furthermore, we observed down-regulation of genes related to translation and neurodegenerative disease, which is of high interest for future studies.

Inhibition of miR-9 resulted in a mild learning impairment in the MWM training phase. In line with these findings, down-regulation of miR-9 has previously been shown in hippocampal neuronal cell culture models stimulated with amyloid-beta peptides, which are known to play decisive roles in memory-related disorders^38^. Decreased miR-9 expression has also been reported in post-mortem samples from several brain regions of Alzheimer’s disease patients^38^,^39^,^40^. In our study, the inhibition of miR-9 led to up-regulation of genes involved in cell adhesion and down-regulation of genes involved in cell death, pointing to roles of miR-9 in axon guidance, synaptic function and neurodegeneration. It has previously been shown in culture that miR-9 inhibition using locked nucleic acid inhibitors (LNAi) causes larger axon lengths and a decreased number of branches per neuron^24^. These highly stable LNAi molecules inhibit specific target miRNAs from exerting their function, similarly to the sponges used in our study. However, a difference from our study is that these LNAi were delivered as a single dose in the experiment, in comparison to our work, where we obtained continuous expression of sponges using AAV vectors. Cellular analysis of axon lengths and dendritic branches was not technically feasible in our setting due to the high density of GFP positive neurons, which increases the difficulty of distinguishing separate sponged neurons in the hippocampus. However, the possibility of a role for miR-9 in axon guidance suggests further investigation *in vivo*.

Lastly, miR-34 inhibition in adult mouse hippocampal neurons resulted in a mild impaired performance in the MWM probe trial, where miR-34sp-injected mice were unable to retain the memory of the platform location. Two previous studies analysing increased levels of miR-34 have implicated this miRNA in ageing and memory function. Firstly, an increased median lifespan was detected after over-expression of this miRNA in *Drosophila*^41^. Secondly, and an increased level of miR-34 in wild-type mice led to impaired associative learning and memory^14^. Our results adds to the complex role of miR-34 in the adult brain.

Our data also show that miR-34 is, in addition to being present in neurons, expressed in glial cells, which may occlude additional information in mRNA-seq data obtained in this work. In our study, the presence of identical miR-34 target genes in glia may partially explain why our neuron-specific inhibition of this miRNA led to milder transcriptome changes in the total mRNA-seq representing the transcriptome of all hippocampal cells, including glia. This is in contrast to the experiments analysing inhibition of miR-124 and miR-9, as these two miRNAs are not expressed in glia, and not in microglia, respectively. Future studies may be employed dissecting the role of miR-34 in specifically hippocampal neurons, as has previously been achieved in other cell types, through cell-specific extraction of RNA^5^. Also, further comparison between the three miRNAs, revealed that shared probable targets with any of the two other miRNAs accounted for 26%, 36% and 46% for miR-124, miR-9 and miR-34, respectively. This means that 46% (26 + 11 + 16 transcripts, see Fig. 3A, right panel) of the probable targets for miR-34 were likely to be influenced by compensatory effects after miR-34 inhibition by miR-124 and miR-9 alone. In addition to all other potentially targeting miRNAs expressed in the hippocampus, this example using three miRNAs visualises the highly complex network through which these regulatory RNAs exert their function upon the transcriptome.

In summary, this study shows the importance of specific miRNAs in hippocampal function. Our findings warrant further studies investigating the implications of these and other miRNAs in neuronal function, and in psychiatric and neurodegenerative disorders.

## Methods

### Adeno-associated viral vectors

The sponge sequences for all vectors used in this work were designed according to^31^, synthesised (Genscript) and cloned into adeno-associated viral (AAV) transfer vectors. The sponge sequences contained eight imperfectly complementary target sites in succession. The viral vectors used in this study were pseudotyped AAV2/5 vectors. The transfer plasmids were cloned using standard techniques. Transgene expression was driven by a human synapsin promoter and all vectors contained a WPRE-element and a late SV40 poly-A sequence. The AAV vectors were produced using a double-transfection method with the appropriate transfer plasmid and the helper plasmid, containing the essential adenoviral packaging genes, as described previously^42^. Vectors were purified by iodixanol step gradients and Sepharose Q column chromatography. The purified viral vector suspension was titrated with TaqMan quantitative PCR and primers targeting the WPRE sequence. The final titers of the injected AAV vector suspensions were between 2.1 × 10^14^ and 4.8 × 10^14^ genome copies/ml.

### AAV injections

All animal-related procedures were approved by and conducted in accordance with the committee for use of laboratory animals at Lund University, Sweden and with the European Communities Council Directive of November 24, 1986 (86/609/EEC). Careful consideration using the three R’s; Replacement, Reduction and Refinement were made to minimise pain, discomfort and stress of the experimental animals. Male C57BL/6 mice of 10 weeks of age were used for AAV sponge injections. Mice were anaesthetised using 2% v/v isofluorane in 0.4 l/min O_2_ and 1.0 l/min N_2_O. Buprenorphine (Temgesic) was applied subcutaneously, followed by intrahippocampal injections of AAV vector suspensions. To this end, four holes were drilled in the skull (two per hemisphere), followed by a total of six 1 μl deposits. The injection coordinates were calculated from bregma, in mm (anteroposterior; mediolateral; dorsoventral from dura): (−2.0;+/−1.4; −1.5), (−2.8;+/−2.7; −1.6) and (−2.8;+/−2.7; −3.7). All vector injections were conducted at 0.4 μl/min using a pulled glass capillary (outer diameter 60–80 μm) that was mounted on a 22-gauge needle and attached to a 5 μl Hamilton syringe. The needle was kept in the brain parenchyma for three minutes after each injection before it was slowly retracted.

### Transgenic mice

Transgenic miR.34.T.GFP sensor mice were generated at École Polytechnique Fédérale de Lausanne through lentiviral transgenesis, as previously described^12^,^17^,^32^. In brief, recombinant lentiviral vectors were injected into the perivitilline space of a fertilised embryo, which was in turn transplanted into a pseudopregnant mouse. The miR-124.T.GFP and miR-9.T.GFP transgenic mice have previously been described^17^,^27^.

### Immunohistochemistry

For immunohistochemical analysis after AAV sponge injection, wild-type mice were transcardially perfused with 4% paraformaldehyde (Sigma), the brains were post-fixed for a minimum of two hours and transferred to 30% w/v sucrose in water. Brains were sectioned coronally on a microtome (35 μm) and put temporarily in phosphate-buffered saline or stored long-term in an antifreeze solution. Standard immunohistochemistry was applied to free-floating sections, as published in detail elsewhere^32^. Primary antibodies used: chicken anti-GFP 1:1000 (Abcam), mouse anti-NeuN 1:1000 (Millipore). The dilution factor of the secondary antibodies was 1:500 (Alexa Fluor, Molecular Probes). Sections stained with 3,3′-diaminobenzidine (DAB) were produced similarly to fluorescence immunohistochemistry, with the addition of initial quenching, the use of a biotinylated peroxidase-bound secondary antibody, incubation in Elite ABC complex (Vector laboratories), followed by incubation in DAB solution and oxidation by hydrogen peroxide.

Adult transgenic miR-34.T.GFP sensor mice were sacrificed through cervical dislocation, the brain tissue was snap-frozen, sectioned and stained using standard immunohistochemistry, as previously described^17^.

### Behavioural testing

All behavioural testing was conducted during daytime and the experimenter was blind for the treatment conditions during all behavioural testing. Starting at seven weeks after vector injection, animals were exposed to the Open Field test (for general activity analysis), followed by the Elevated plus maze (for anxiety-like behavioural analysis), with one day of resting between the tests. The following week, the Morris water maze (MWM) test was used to evaluate spatial learning and memory. Additionally, a custom-made closed plus maze was used to test working memory capacity in a spontaneous alternation task (SAT). For further details on behavioural testing, see Supplementary Methods.

### RNA preparation and mRNA-sequencing

Ten weeks after injection, bilateral hippocampal tissue was quickly dissected from decapitated AAV vector-injected mice, snap-frozen and homogenised in Buffer RLT with 0.01% v/v β-merkaptoethanol in a TissueLyserLT at 50 Hz for two min. Total RNA was extracted using the RNeasy mini kit, following instructions of the supplier (Qiagen). A total of 12 mice were used for mRNA-seq. For each miRNA experiment (miR-9 and miR-34), we used three mice injected with AAV-GFP control (GFP Ctrl) and three mice injected with either AAV-GFP-miR-9sp or –miR-34sp. cDNA libraries of mRNA samples were prepared using the NuGEN Ovation RNA-Seq System including poly-A enrichment and Illumina high-throughput sequencing was applied to the samples by Clinical Microarray Core (UCLA, CA, USA). The raw sequencing data for the miR-124 experiment was previously described^8^.

### RNA analysis

In mRNA-seq, the total number of input reads (after trimming) were 252 246 253 (miR-9) and 153 793 712 (miR-34), respectively. The 50 bp single end reads were mapped to the mouse genome (mm9) and visualised in the UCSC genome browser. Reads were quantified to Refseq. The data was scaled according to the average read number per sample and these scaled values were used to analyse changes in RNA levels.

Gene ontology analysis was conducted using the online DAVID bioinformatics database tool (http://david.abcc.ncifcrf.gov). Genes with a read number >10 in the mRNA-seq data of GFP Ctrl mice in each respective miRNA experiment, were used in a background list for the functional annotation analysis of each data set. Using medium default stringency, we identified all (or the top 10 most highly) significantly enriched (p < 0.05) Gene Ontology biological processes (GO_BP) and Kyoto Encyclopedia of Genes and Genomes (KEGG) pathways in a functional annotation chart. KEGG pathways were labelled in bold font. For comparison between different miRNAs, any shared significant GO_BPs and KEGG pathways were visualised as stacked bars.

IPA was performed using all genes with a read number >10 across all samples of each of the three miRNA sponge experiments. The RefSeq ID, log2(average fold change in miRNA sponge/GFP Ctrl) and p-value (unpaired parametric t-test) were uploaded and a Core analysis was performed using standard settings with Refseq genome as ‘MouseRef-8 v2.0’, Confidence as ‘Experimentally observed’ and ‘High (predicted)’, in mammals (human, mouse, rat), ‘Tissues & Cell Lines’ as ‘Hippocampus’, with ‘Up/Downregulated genes’ using cut-offs log ratio as 0.2630344 and p-value <0.05.

Subsets of target mRNAs for each miRNA family were identified using different factors. In subsets containing probable miRNA targets, all genes had previously been identified as mRNAs present in the RISC of hippocampal neurons^8^. Furthermore, all genes in these subsets had at least one computationally predicted and evolutionary conserved target site for each respective miRNA (TargetScanMouse 6.2). Another subset (independent of identified probable targets) contained previously validated target genes for miR-124 using miRWalk 2.0^43^ and TarBase 7.0^44^.

The same RNA samples used for mRNA-seq were analysed using an Affymetrix microarray (SciBlu genomics). Significantly up- and down-regulated genes and affiliated GO_BPs and KEGG pathways in both datasets were compared. The 21 most up- and down-regulated genes in RNA-seq that were also detected in the microarray dataset were visualised.

### Statistical Analyses

Data was depicted in text and figures as mean+/− standard error of the mean (SEM), unless otherwise specified. Unpaired parametric two-tailed t-tests were used to analyse means of two groups. Two-way analysis of variance (ANOVA) with repeated measures and Sidak’s multiple comparisons test was used to calculate significant differences in the MWM training phase. Kolmogorov-Smirnov Z tests were used to determine significant differences between two cumulative fraction graphs. Fischer’s Exact test was used to calculate the p-value of data originating from IPA. The criterion for significance and thresholds for all analyses was p < 0.05.

## Additional Information

**Accession Numbers:** RNA-seq data was deposited in the Gene Expression Omnibus database at the NCBI under the accession number GSE68884.

**How to cite this article**: Malmevik, J. *et al.* Distinct cognitive effects and underlying transcriptome changes upon inhibition of individual miRNAs in hippocampal neurons. *Sci. Rep.* **6**, 19879; doi: 10.1038/srep19879 (2016).

## Supplementary Material

Supplementary Information

Supplementary Table S1

Supplementary Table S2
