# Supplementary material for: Distinct cognitive effects and underlying transcriptome changes upon inhibition of individual miRNAs in hippocampal neurons
Source: Sci Rep. 2016 Jan 27;6:19879. doi: 10.1038/srep19879 (PMC4728481; doi:10.1038/srep19879)
Supplement: Supplementary Information [file srep19879-s1.pdf]

Distinct cognitive effects and underlying transcriptome changes upon inhibition of individual miRNAs in hippocampal neurons

Josephine Malmevik, Rebecca Petri, Pina Knauff, Per Ludvik Brattås, Malin Åkerblom and Johan Jakobsson

Supplementary Fig. S1

Supplementary Fig. S2

Supplementary Table S1

Supplementary Table S2

Supplementary Methods

Supplementary Fig. S1: Further analysis of validated targets, probable targets and microarray data

A) The 21 most down-regulated (grey) and 21 most up-regulated genes (red in upper panel, purple in bottom panel) after miR-124sp injection as shown using RNA-seq data (upper panel) and microarray data analysis (lower panel). B) For all significantly up-regulated and down-regulated genes after miR-124 inhibition, gene ontology analysis was compared in RNA-seq data (upper panel) with that of microarray data (lower panel). Related to Fig. 3-4.

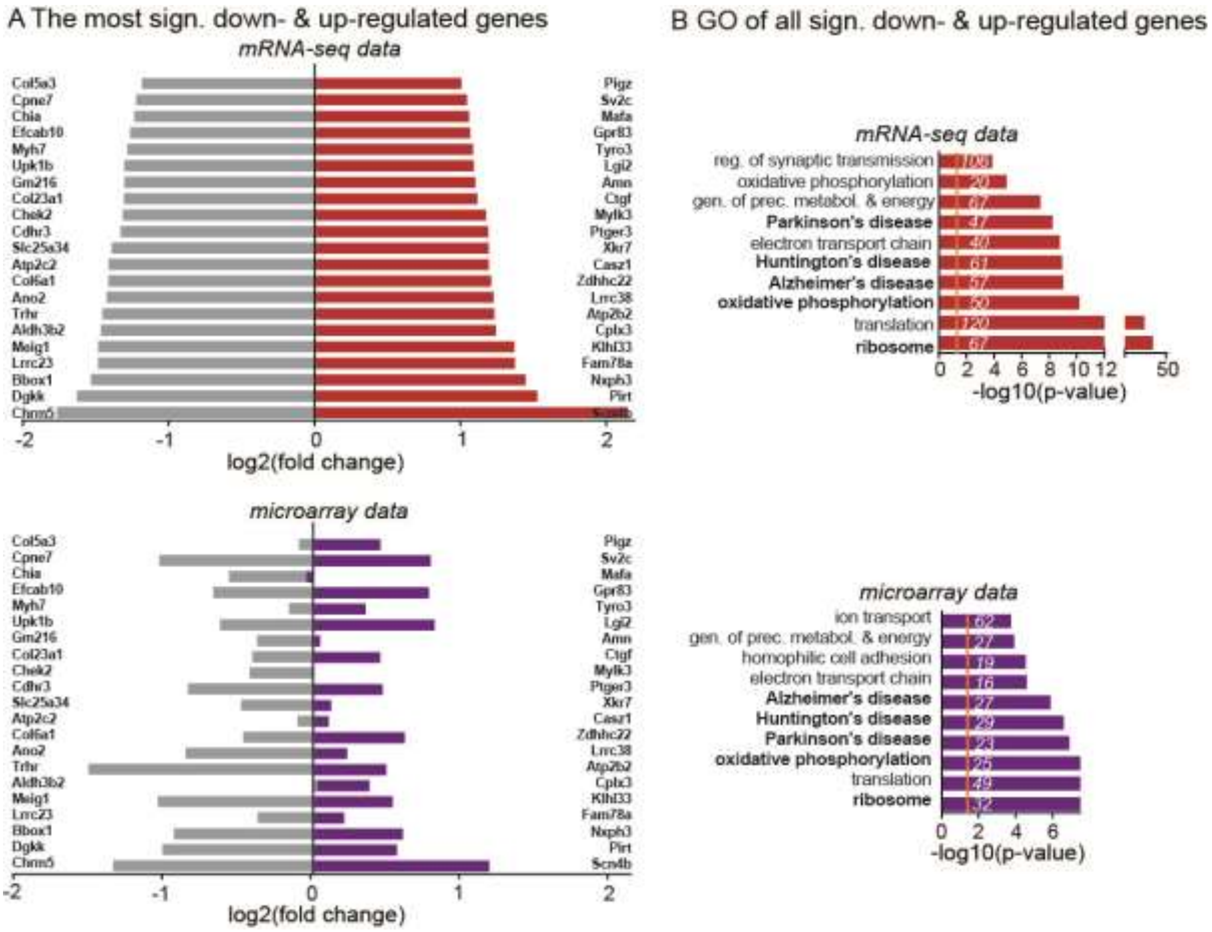

Supplementary Fig. S2: miRNA inhibition does not affect general activity or anxiety-like behaviour

At the end of MWM testing, the outcome of a visible platform (VPF) test ensured equal motivation and locomotor ability in all experimental groups with regards to the miR-124sp (A), miR-9sp (E), and miR-34sp (I) experiments. The spontaneous alternation in a SAT task was not due to differences in general activity in this plus-shaped maze for either miR-sp experiment (B,F,J, respectively). No difference was detected in general activity (Open Field; C, G, K, respectively) or anxiety-like behaviour (Elevated plus maze; D, H, L, respectively). Related to Fig. 6.

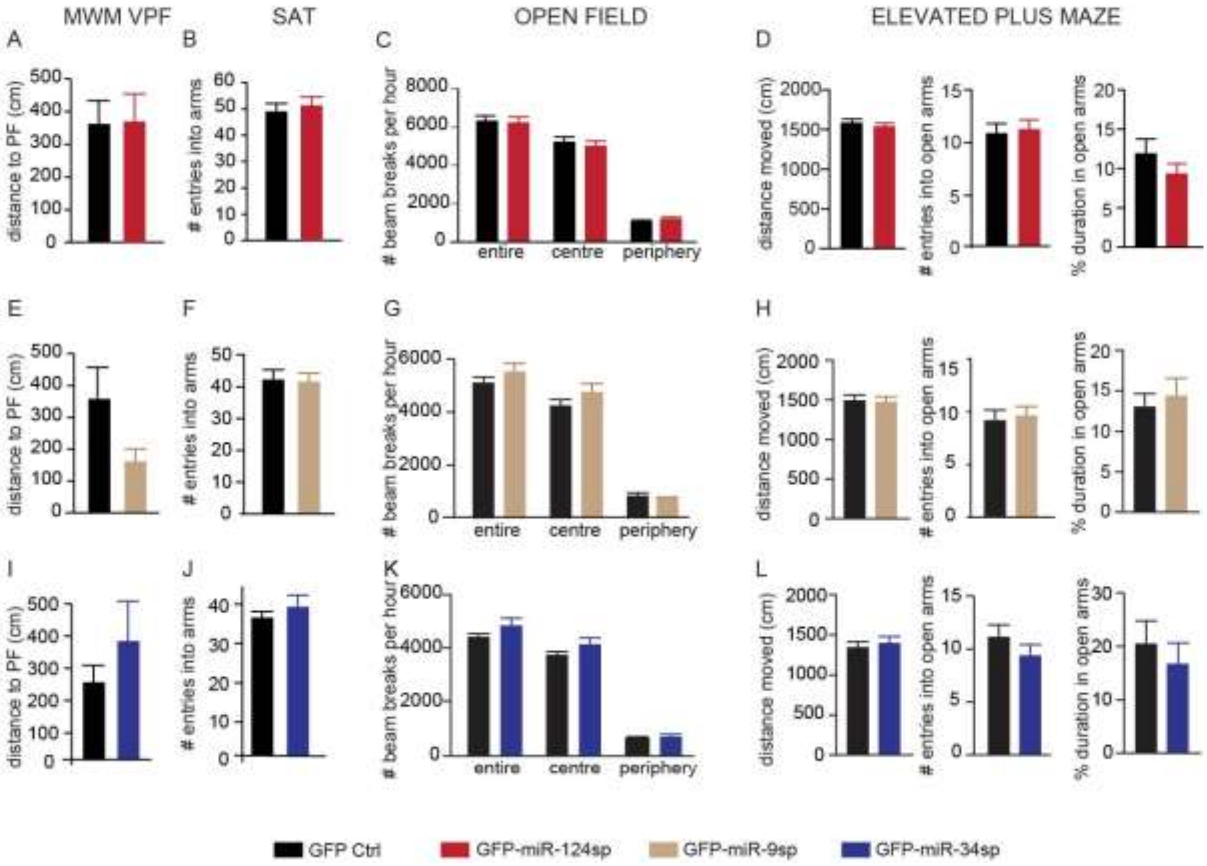

Supplementary Table S1: Validated target genes up-regulated after miR-124 inhibition

Inhibition of miR-124 resulted in up-regulation of already validated targets (according to miRWalk and tarbase databases). Related to Fig. 3.

Supplementary Table S2: Ingenuity Pathway Analysis reveals specific transcriptome changes after the inhibition of each miRNA

The three miR-sp experiments (miR-124sp, miR-9sp, miR-34sp) resulted in different patterns of Canonical pathways (A), Upstream regulators (B), and Diseases and Bio Functions (C), visualised in a separate data set (excel file) on a –log10(p-value) scale. Related to Fig. 5.

Supplementary Methods

To eliminate odour cues, each apparatus was thoroughly cleaned with 70 % ethanol and dried after each animal. General motor activity in the Open Field task was assessed in a white-floored box (50 x 50 x 37 cm) with transparent walls under dim and dispersed light conditions. Two adjacent sides contained rows of beams, forming a coordinate system, connected to a data-processing computer. Each mouse was placed in the centre of the field and allowed unimpeded exploration for 60 min. The total amount of line crossings of all four paws was captured via beam breaks (PASdata), which was used as an index of motor activity.

The Elevated plus maze test was used as a test for anxiety-like behaviour. This maze consisted of four arms (each 66 x 6 x 14 cm); two opposite open arms (without walls) and two opposite closed arms (with walls), arranged at perpendicular angles. The arms extended from a common central platform (6 x 6 cm), giving equal access to all arms. The maze was elevated 70 cm above the floor under dim and dispersed light conditions. Mice were placed on the central platform and allowed unimpeded exploration for five min, automatically recorded by the video tracking software (Ethovision 3.1.16, Noldus).

In the MWM test, a large circular pool (180 cm diameter) filled with opaque water (19°C +/- 1°C). A platform (15 cm diameter) was submerged 10 mm under the water surface. The water maze was surrounded by a white periphery with specific distal visual cues, pseudo-randomly positioned around the pool. White noise was produced from a radio centrally positioned above the pool to impede the use of auditory cues for navigation. One day before the learning trials began, all mice were habituated via a two min swim trial without a platform, where the mouse was placed in the middle of the pool. Spatial learning sessions were conducted on the following three consecutive days with four trials per day and an inter-trial interval of two hours. Each trial was started by introducing the mouse, facing the pool wall, at one of four starting points, pseudo-randomly counterbalanced between trials and days. The distance moved to reach the platform was measured as an index of the learning and memory capacity of the animals. If a mouse did not find the platform within 60 s, it was gently guided to the platform. Each mouse remained on the platform for 30 s before transfer to a heated waiting cage. During all learning trials, the platform remained in the same position. Averages for paired trials were organised

into blocks (B1, B2, etc) for analysis. On the day following the last learning trial, a 30 s probe test was conducted, where the platform had been removed from the pool. The percentages of the duration spent in the target and opposite quadrants were calculated, and served as an indication of a retained spatial reference memory. A visible platform test was conducted at the end of the experiment to ensure that the motivation and locomotor ability was not significantly different between the mice of the experimental groups. This involved the positioning of a visible object on top of the platform. The plus-shaped maze was composed of four arms (each 40 x 8 x 14 cm) joined to a central square platform of eight cm width. SAT testing was conducted by placing the mouse on the centre platform and allowed 10 min of unimpeded exploration. The number and sequence of arm entries were manually recorded to calculate the number of alternations. A full alternation consisted of at least one instance of all four possible arm choices in five consecutive arm entries. The number of observed full alternations in overlapping quintuplets was divided by the number of maximum possible alternations, and the quotient was multiplied by 100 to get the final SAT score.
